# Enhanced Performance of Inverted Non-Fullerene Organic Solar Cells by Using Metal Oxide Electron- and Hole-Selective Layers with Process Temperature ≤150 °C

**DOI:** 10.3390/polym10070725

**Published:** 2018-07-02

**Authors:** Hailong You, Lin Dai, Qianni Zhang, Dazheng Chen, Qubo Jiang, Chunfu Zhang

**Affiliations:** 1 State Key Discipline Laboratory of Wide Band Gap Semiconductor Technology, School of Microelectronics, Xidian University, 2 South Taibai Road, Xi’an 710071, China; hlyou@mail.xidian.edu.cn (H.Y.); 15249296604@163.com (L.D.); qiannizhang93@outlook.com (Q.Z.); dzchen@xidian.edu.cn (D.C.); 2 School of Electronic Engineering and Automation, Guilin University of Electronic Technology, No. 1 Jinji Road, Guilin 541004, China; boblincoln@sina.com

**Keywords:** organic solar cells, non-fullerene acceptor, electron-selective layer, aqueous solution

## Abstract

In this work, an efficient inverted organic solar cell (OSC) based on the non-fullerene PBDB-T:IT-M blend system is demonstrated by using an aqueous solution processed ZnO electron-selective layer with the whole process temperature ≤150 °C and a thermally evaporated MoO_3_ hole-selective layer The ZnO selective layer is deposited by aqueous solution and prepared in a low-temperature process, so that it can be compatible with the roll-to-roll process. The proposed device achieves an enhanced power conversion efficiency (PCE) of 9.33% compared with the device based on the high-temperature sol-gel-processed ZnO selective layer, which achieves a PCE of 8.62%. The inverted device also shows good stability, keeping more than 82% of its initial PCE after being stored under ambient air conditions and a humidity of around 40% without any encapsulation for 240 h. The results show the potential for the fabrication of efficient non-fullerene OSCs with low-temperature metal oxide selective layers.

## 1. Introduction

Organic solar cells (OSCs) have become one of the most promising candidates for renewable energy sources due to their advantages of low cost solution process capability. For the conventional OSCs, although the absorption ability of fullerene is inferior, PC_61_BM and PC_71_BM, which are fullerene derivatives, still play an important role as electron acceptors in polymer-fullerene blend systems [1,2]. In the past few years, non-fullerene OSCs, including non-fullerene polymers or small molecules as the electron acceptor, have attracted tremendous interest for further improving the power conversion efficiency (PCE) of OSCs due to their advantages of strong optical absorption, easily tunable energy levels, high crystalline properties, and so forth [3,4,5,6,7,8]. Subsequently, many efforts have been focused on the molecular structure of fullerene-free acceptors, donor-acceptor matching criteria, and quantifying interaction-function relations to achieve efficient devices [9,10,11]. So far, the efficiency of non-fullerene OSCs has been promoted from 9% to 12%, demonstrating the huge potential of fullerene-free acceptors in OSCs [12,13,14,15,16,17,18,19].

Besides the active materials, the device structure is also important. In general, there are two main structures of OSCs: the conventional structure and the inverted structure. The difference between the two structures is the sequence of electrodes. In many conventional OSCs, an acidic poly(3,4-ethyl-enedioxythiophene):poly(styrene sulfonate) (PEDOT:PSS) on an indium tin oxide (ITO) electrode is usually selected as the hole-selective layer and low work function metals such as aluminum (Al) or calcium (Ca) are used as the top electrode. However, the ITO electrode can be corroded by the PEDOT:PSS layer and the top metal electrode can be easily oxidized in air, both resulting in poor stability of the cells [20,21,22,23,24,25,26,27]. To improve the stability of OSCs, the inverted structure is widely used [25,26,27,28,29,30]. In the inverted structure, the ITO with an n-type metal oxide film such as ZnO or TiO_2_ is used as the cathode selective layer to collect the electrons and high work function metals such as silver (Ag) or gold (Au) with MoO_3_ are used as the top anode to collect holes. Furthermore, the acceptor/donor blend system is protected by two metal oxide carrier selective layers, which can enhance the stability of OSCs.

In the inverted structure, key points in fabricating efficient OSCs, including single OSCs and tandem OSCs, are the material selectivity of the electron selective buffer layer and interface modification of the inverted structure [31,32,33]. These materials used in the cathode electron-selective buffer layer ought to have high electron mobility and at the same time exhibit good hole-blocking capability. Many efforts have been devoted to improving the performance of inverted OSCs by using the ZnO film as the electron-selective layer, owing to its high electron mobility, ohmic contact with an active layer, and good optical transmittance. The methods of making solution-processed ZnO, such as sol-gel, nanocrystalline ZnO (nc-ZnO) particles, and aqueous-solution routes, are very attractive and have been investigated extensively [27,29,34]. However, the sol-gel method employs a high-temperature (usually >250°) process, which may be not compatible with flexible substrates, and the electrical performance of nc-particle-processed ZnO is sensitive in ambient air. Recently, the technique of ammine-zinc complex aqueous-solution-processed ZnO has demonstrated promising potential, which can solve these problems and acquire a dense ZnO film at a low temperature [21,35]. This method of depositing a ZnO film was first adopted by Meyer et al. to fabricate thin film transistors [36]. Afterward, a similar way of depositing ZnO as the electron-selective buffer layer was introduced in organic light-emitting diodes and inverted OSCs [21,25,37,38]. Particularly, inverted OSCs which are based on ZnO deposited by this method show promising performance [39,40]. However, this method has never been used in non-fullerene OSCs.

In this work, we have employed the aqueous-solution-processed ZnO film as the electron-selective layer and the thermally evaporated MoO_3_ film as the hole-selective layer in inverted OSCs based on a non-fullerene material system with the whole process temperature ≤150 °C. Here, a small-bandgap material named poly[(2,6-(4,8-bis(5-(2-ethylhexyl)thiophen-2-yl)benzo[1,2-b:4,5-b′] dithiophene)-*co*-(1,3-di(5-thiophene-2-yl)-5,7-bis(2-ethylhexyl)-benzo[1,2-c:4,5-c′]dithiophene-4,8-dione)] (PBDB-T) is used as the donor and a non-fullerene material named IT-M is used as the acceptor [16,41]. In the devices, the low-temperature-processed ZnO could be compatible with the roll-to-roll process. At the same time, both the donor and the acceptor could absorb solar light in this material system and have the potential to demonstrate high photovoltaic performance in devices. With the configuration of glass/ITO/ZnO/PBDB-T:IT-M/MoO_3_/Ag, an efficient inverted OSC with a PCE of 9.33% is fabricated. The inverted device also shows good stability, which could maintain 82% of its initial PCE after 240 h storage when exposed under ambient air conditions and a humidity of around 40% without any encapsulation.

## 2. Experimental Section

### 2.1. Materials and Reagents

For the aqueous precursor solution, we dissolved ZnO powder (99.9%, particle size <5 µm, Sigma-Aldrich, Saint Louis, MI, USA) in ammonia (25%, Tianjin Chemical Reagent, Tianjin, China) to form a 0.1 M Zn(NH_3_)_4_^2+^ solution, then the solution was refrigerated for more than 12 h after being ultrasonically processed for 10 min. For the sol-gel solution, we dissolved zinc acetate dehydrate Zn(CH3COO)2·2H_2_O (99.9%, Aldrich) in anhydrous ethanol CH3CH2OH (99.5%, Aldrich) and added ethanolamine as the stabilizer followed by stirring for over 12 h at 60 °C. PBDB-T and IT-M were purchased from Dalian Lichuang Chemical Co., Ltd., Dalian, China. PBDB-T and IT-M were dissolved in a chlorobenzene/1,8-diiodoctane (99:1 vol %) mixed solvent with a total concentration of 20 mg/mL and 1:1 weight ratio. The solution of PBDB-T and IT-M was stirred at 100 °C for 12 h before use. ITO-coated glass substrates have a sheet resistance about 10 Ω/sq. The chlorobenzene and MoO_3_ were supplied by Sigma-Aldrich. All the materials were used without any further purification.

### 2.2. Fabrication of Solar Cells

**Figure 1:**
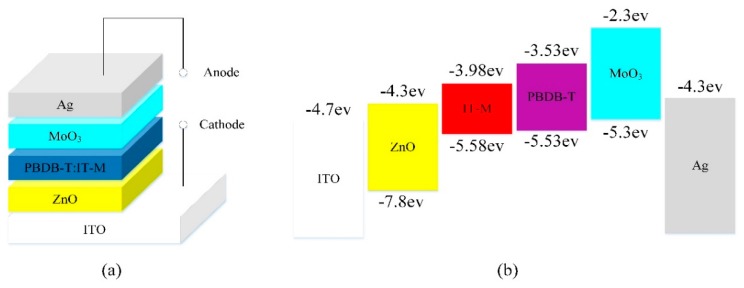
(**a**) Schematic device structures of inverted organic solar cells (OSCs); (**b**) energy level alignment of the components in the inverted OSCs with ZnO as the electron transport layer.

The inverted devices based on PBDB-T:IT-M have been prepared in this work. As shown in Figure 1a, the devices have the configuration of glass/ITO/ZnO/PBDB-T:IT-M/MoO_3_/Ag and they were fabricated on patterned ITO-coated glass substrates. ITO-coated glass substrates were cleaned by a routine solvent ultrasonic cleaning, sequentially with detergent (Decon 90, Hove, UK), deionized water, acetone, and ethanol in an ultrasonic bath for about 20 min, and then were blown dry by a nitrogen gun (Kewatt, Zhongshan, China) before use. Next, the ITO-coated glass substrates were treated by UV ozone for 15 min. Following that, a layer of ZnO, using ZnO aqueous solution, was spin-coated on cleaned ITO glass substrates at 3000 rpm for 45 s and then annealed on a heating stage at 150 °C for 15 min under ambient conditions. For comparison, the devices with the ZnO layer spin-coated by sol-gel method at 3000 rpm for 60 s were also fabricated, and then annealed at 275 °C for 15 min. The PBDB-T:IT-M solution was spin-coated on the ZnO films at 3000 rpm for 120 s to obtain the optimal film thickness of ≈100 nm in a nitrogen-filled glove box, followed by annealing in N_2_ at 150 °C for 30 min. Finally, 8 nm MoO_3_ and 100 nm Ag were deposited via a thermal evaporation method through a metal shadow mask in a vacuum chamber. The device area of the solar cells is 70 mm^2^.

### 2.3. Characterization

All the current density–voltage (J–V) characteristics of devices were obtained under a Keithley 2400 source measure unit (Tektronix, Inc., OR, USA) and a Xenon lamp with an AM 1.5G filterXES-301, SEN-EI Electric. Co. Ltd, Osaka, Japan). All the Incident photo-to-electron conversion efficiency (IPCE) was measured through a quantum efficiency measurement system (IPCE, Zolix Instruments Co., Ltd., Beijing, China). The thin films morphology was gained through the tapping mode atomic force microscopy (AFM) tests by an Agilent 5500 scanning probe system ((AFM, Bruker Dimension Icon, Bruker, Karlsruhe, Germany). The transmittance spectra were investigated by UV–Vis/NIR spectrophotometer (Perkin-Elmer Lambda-950, Waltham, MA, USA). The photoluminescence (PL) spectrum was also utilized to analyze the ZnO thin film using the 325 nm line of a He–Cd laser (FLS980, Edinburgh, United Kingdom).

## 3. Results and Discussion

The corresponding energy band diagram of the inverted OSCs is illustrated in Figure 1b. The conduction band energy of pure ZnO is around −4.4 eV and the valence band energy of pure ZnO is around −7.8 eV, which suggests that electrons from IT-M can be transported into ZnO, while holes from PBDB-T can be blocked. In other words, ZnO serves as an electron-selective layer and a hole-blocking layer. Meanwhile, MoO_3_ acts as the role of both the hole-selective layer and the electron-blocking layer and can effectively enhance the transportation of holes into the anode.

**Figure 2:**
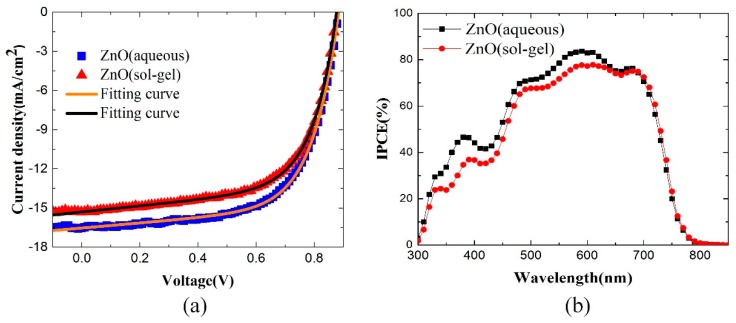
(**a**) J–V characteristics of inverted OSCs with the ZnO selective layer using aqueous or sol-gel under the simulated AM 1.5G illumination of 100 mW/cm^2^; (**b**) the IPCE curves of PBDB-T:IT-M OSCs.

The obtained light J–V curves of the inverted OSCs, which are tested under AM 1.5G illumination with 100 mW/cm^2^ light intensity, are shown in Figure 2a. The J–V characteristics of OSCs can be approximately described by the Shockley equation:

(1) $$J=J_{0}\left\{\exp\left[\frac{q(V-R_{s}J)}{nk_{B}T}\right]-1\right\}+\frac{V-R_{s}J}{R_{sh}}-J_{ph}$$

where *J*_0_ is the saturation current, *J_ph_* the photocurrent, *R_s_* the series resistance, *R_sh_* the shunt resistance, *n* the ideality factor, *q* the electron charge, *k_B_* the Boltzmann constant, and *T* the temperature, respectively. By combining Equation (1) with the proposed explicit analytic expression method [42], the experimental data can be well reconstructed as shown in Figure 2a, which confirmed the validity of the extracted parameter, and the extracted photovoltaic parameters are also displayed in Table 1. The non-fullerene OSC with the ZnO layer deposited by the aqueous solution in this work shows a best PCE of 9.33%, with an open-circuit voltage (*V*_OC_) of 0.88V, a short current density (*J*_SC_) of 16.54 mA/cm^2^, and a fill factor (FF) of 63.84%. For the comparison, the device with the ZnO layer spin-coated by the sol-gel solution achieves a best PCE of 8.62% with a *V*_OC_ of 0.87 V, a *J*_SC_ of 15.30 mA/cm^2^, and a FF of 64.35%. By comparing the devices with the ZnO layer deposited by the aqueous solution and the sol-gel solution, there are no obvious differences for *V*_OC_ and FF. The device with the ZnO layer deposited by aqueous solution has a relative high *J*_SC_ when compared with that deposited by the sol-gel method. Furthermore, the enhanced *J*_SC_ results in an improved PCE for the the device with the ZnO layer deposited by aqueous solution, as shown in Figure 2a. It should be noted that the main difference between the two devices is the carrier selective layer. In addition, the annealing temperature of the ZnO layer spin-coated by the aqueous solution method is far lower than that of the devices deposited by the sol-gel method, so the inverted non-fullerene OSCs with the aqueous-solution-processed low-temperature ZnO selective layer have the potential to be applied in flexible devices. Our recent research results have demonstrated that the inverted flexible OSCs with the aqueous-solution-processed ZnO layer show promising performance in polymer-fullerene blend systems [31], and the results here show that it is still valid in the non-fullerene systems.

For further comparison, the IPCE curves of the devices based on aqueous solution and sol-gel solution are shown in Figure 2b. Both of the devices show high quantum efficiencies at the wavelength coverage of 400–750 nm. The device based on aqueous solution presents the maximum IPCE of 83.6%, higher than the maximum value 77.8% of the device based on sol-gel solution, which can be attributed to the film quality of ZnO. The integrated *J*_SC_ based on the IPCE curve is 16.14 mA/cm^2^ for the ZnO using aqueous solution and 15.31 mA/cm^2^ for the ZnO using sol-gel solution, respectively; only slightly lower than their measured *J*_SC_ (16.54 and 15.30 mA/cm^2^). The devices degradatedduring the IPCE test in the air atmosphere can probably explain this result. Anyway, the IPCE measurement results again confirm that the device based on aqueous solution has a higher *J*_SC_ than that based on sol-gel solution.

**Figure 3:**
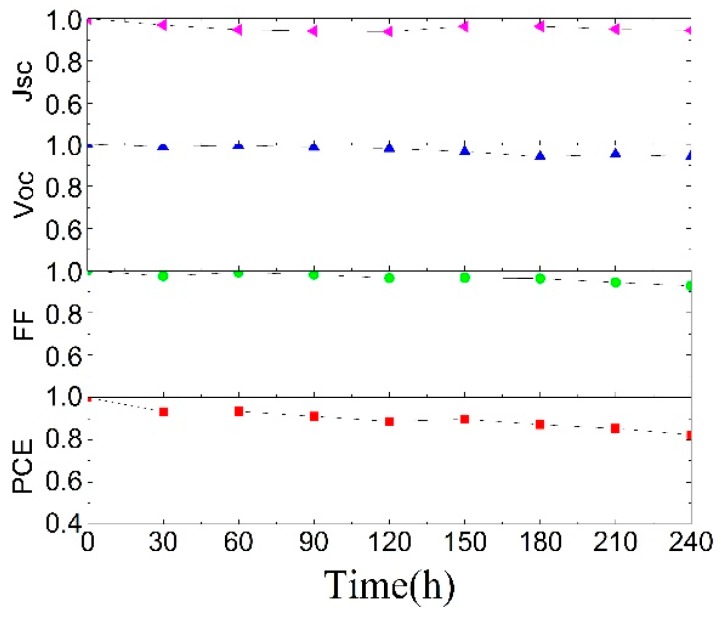
Parameter variations of the inverted devices at different storage times in air. The tests were conducted under ambient air conditions and a humidity of around 40% without any encapsulation.

Besides the PCE, the stability of the inverted OSCs with the aqueous-solution-processed ZnO layer is also investigated. We stored these devices under ambient air conditions and a humidity of around 40% without any encapsulation. The variations of the performance parameters at different storage times are shown in Figure 3. As time passes, *V*_OC_ and FF change slowly. After 240 h storage, the PCE of the inverted OSCs still remains above 82% of its initial value, which exhibits that the inverted device has a high stability.

**Figure 4:**
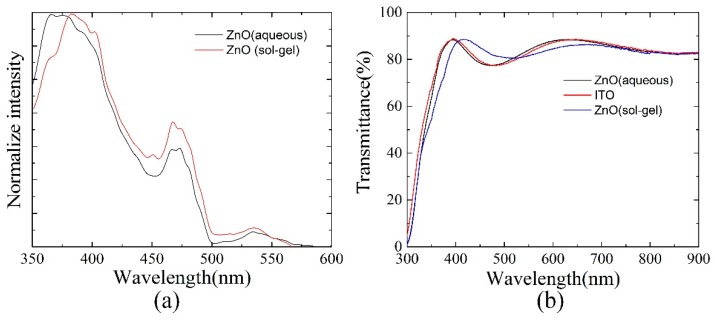
(**a**) Photoluminescence spectra of the ZnO film using aqueous solution or sol-gel solution methods under 325-nm light excitation, respectively; (**b**) transmittance spectra of ITO, ITO/ZnO(aqueous), and ITO/ZnO(sol-gel).

The photoluminescence measurement was adopted to characterize the properties of ZnO films. Figure 4a shows the PL spectra of the ZnO layer using aqueous solution after annealing at 150 °C for 15 min in ambient air and the ZnO layer using sol-gel solution after annealing at 275 °C for 15 min in ambient air, under excitation of 325-nm light. It is obvious that the sharp ultraviolet (UV) emission peak is at around 370 nm for the ZnO layer deposited by aqueous solution and the UV emission peak is at around 380 nm for the ZnO layer spin-coated by sol-gel method. In common, a broad visible emission peak around 520 nm is also observed in the ZnO PL spectrum, which is usually associated with singly ionized oxygen vacancy in ZnO film, resulting in radiative recombination of a photogenerated hole with an electron occupying the oxygen vacancy [43]. Here, we can find that the green emission peaks of all the ZnO films are very weak, showing that defects in each of the samples are in a low level [44]. If comparing the two samples carefully, the green peak of the ZnO film deposited by aqueous solution is smaller than that spin-coated by sol-gel method. It means that although there is a much lower annealing temperature for the ZnO film deposited by aqueous solution than that spin-coated by sol-gel method, it still could achieve a high quality. The transmittance spectra of ZnO thin films are shown in Figure 4b. Obvious oscillation is observed for all the samples. Except for the different oscillation peaks, there is no obvious difference in transmittance spectra for the samples made using either aqueous solution or sol-gel solution. Average transmittance value ~83% in visible range is recorded for two samples, both ensuring more photon flux at the active layer.

**Figure 5:**
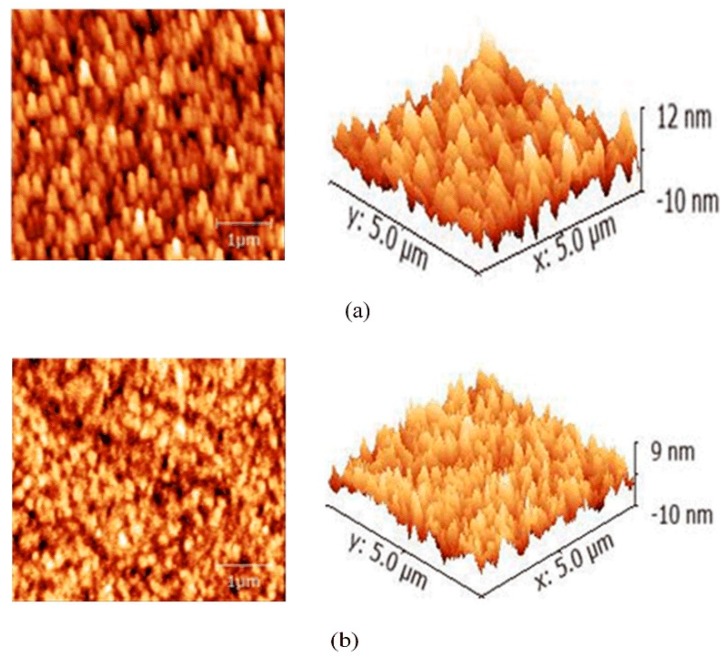
(**a**) Surface morphologies of (**a**) ITO/ZnO (aqueous) and (**b**) ITO/ZnO (sol-gel).

AFM was adopted to characterize the morphology of the ZnO films. Figure 5 shows the results. It is found that the ZnO layer deposited by aqueous solution has the largest Root-Mean-Square (RMS) of 2.95 nm, while the RMS of the ZnO layer spin-coated by sol-gel method is about 2.51 nm. Both are relatively smooth, which lays the foundation of obtaining the ultimate device performance.

**Figure 6:**
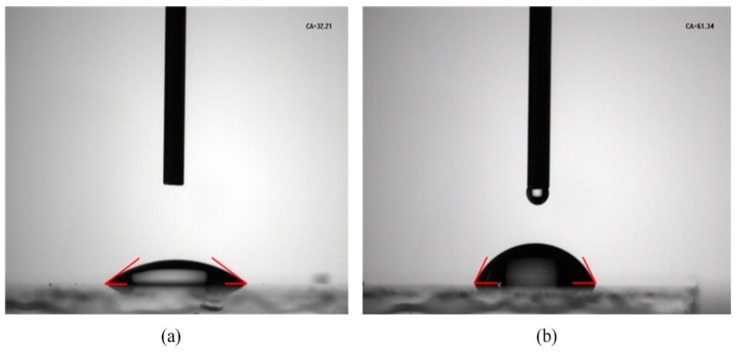
Contact angle (CA) measurements of (**a**) ZnO (aqueous) and (**b**) ZnO (sol-gel).

Contact angle measurement was carried out to detect hydrophilicity of the ZnO films, which will affect the formation of the active layer. Generally, if the contact angle of a material is less than 90°, then we consider it to be hydrophilic, whereas if the contact angle is greater than 90°, we consider it to be hydrophobic. The smaller the contact angle, the better the hydrophilicity. The result is shown in Figure 6. The contact angle of water is 32.21° for the ZnO layer deposited by aqueous solution, which is lower than the contact angle of 61.34°for the ZnO layer using sol-gel method. This shows a better hydrophilicity of the ZnO film using the aqueous solution, which is one reason for the better device performance.

## 4. Conclusions

OSCs based on PBDB-T:IT-M were fabricated with low-temperature-processed ZnO and MoO_3_ carrier selective layers in this work. The low-bandgap material PBDB-T is employed so that more solar light can be harvested by the device; the non-fullerene acceptor named IT-M is adopted to enhance the absorption ability of the Acceptor/Donor system; and a ZnO selective layer is deposited by aqueous solution and prepared in a low-temperature process, so that it can be compatible with the roll-to-roll process. The proposed device achieves an enhanced PCE of 9.33% compared with the device based on the sol-gel-processed ZnO selective layer, which achieves a PCE of 8.62%. The inverted device shows good stability, keeping more than 82% of its initial PCE after being stored for 240 h. The result shows a promising potential for the fabrication of efficient, flexible non-fullerene organic solar cells with low-temperature metal oxide selective layers.

## Figures and Tables

**Table 1:** Performance parameters of inverted OSCs on glass substrates.

Device	*V*_OC_^a^ (V)	*J*_SC_^a^ (mA/cm^2^)	FF ^a^ (%)	PCE ^a^ (%)	PCE_max_ (%)	*J*_ph,max_ (mA/cm^2^)	*J*_0,max_ (mA/cm^2^)	*n*_max_	*R_s_*,_max_ (Ω.cm^2^)
ZnO (aqueous)	0.87 ± 0.01	16.07 ± 0.39	63.12 ± 0.65	8.91 ± 0.23	9.33	16.55	3.03 × 10^−4^	3.16	6.17
ZnO (sol-gel)	0.86 ± 0.01	15.13 ± 0.12	63.13 ± 0.89	8.36 ± 0.15	8.62	15.32	3.14 × 10^−5^	2.62	5.53

^a^ Average values are obtained from over 10 devices. ^b^ The subscript “max” means the parameters of the best-performing device.
